# Mycobacteria Infection in Incomplete Transverse Myelitis Is Refractory to Steroids: A Pilot Study

**DOI:** 10.1155/2011/501369

**Published:** 2011-02-07

**Authors:** Yanqing Feng, Ning Guo, Junxiu Liu, Xi Chen, Qiaosong Sun, Rong Lai, Fan Huang

**Affiliations:** Department of Neurology, The First Affiliated Hospital of Sun Yat-sen University, 2 Road 58 Zhongshan, Guangzhou 510080, China

## Abstract

Incomplete transverse myelitis (ITM) of unknown origin is associated with high rates of morbidity and mortality. This prospective, open-label study was undertaken to determine whether antituberculous treatment (ATT) might help patients with ITM whose condition continues to deteriorate despite receiving IV methylprednisolone treatment. The study consisted of 67 patients with steroid-refractory ITM in whom *Mycobacterium tuberculosis* (MTB) was suspected clinically and in whom other known causes of myelopathy were excluded. The study occurred from January 2003 to June 2010. Patients underwent trial chemotherapy with ATT. Efficacy was assessed by the American Spinal Injury Association (ASIA) scoring system, the Barthel Index (BI) and the Hauser Ambulation Index (AI) at baseline, 12 months, and 24 months, using magnetic resonance imaging (MRI). Of the 67 patients enrolled, 51 were assessed and 16 withdrew. At 24 months, 49 patients experienced benefits as indicated by significantly increased ASIA and BI scores. The Hauser AI index also improved with markedly decreased abnormal signals in spinal cord MRI over time. The results from this prospective study provide beneficial clinical and MRI data on the efficacy of ATT in ITM patients and suggests mycobacteria may be an important and neglected cause of myelitis.

## 1. Introduction

Transverse myelitis is a focal inflammatory disorder of the spinal cord, resulting in motor, sensory, and autonomic dysfunction. It is a poorly defined condition and displays heterogeneous etiopathogenesis [1, 2]. Incomplete transverse myelitis (ITM) is a rare syndrome easily recognized in clinical practice, with symptoms and signs producing bilateral partial impairment of motor, sensory, and autonomic functions, usually with an acute or subacute onset. Inflammation is not only the commonest basis of ITM but is also the most difficult to identify [3]. This clinical picture can be shared by variety of etiologies including different bacterial or viral infections. Clinical presentations of infectious and demyelinating diseases of the spinal cord are protean and often nonspecific. Magnetic resonance imaging (MRI) is sensitive but lacks specificity and most of the lesions feature high signal intensity on T2-weighted images. There does not seem to be any clinical, immunological, or radiological strategies that reliably distinguish between demyelinating, infectious, inflammatory, vascular, neoplastic, or paraneoplastic etiologies. Therefore, physicians must be aware of the many potential treatable etiologies for ITM which might mean there exists an efficient approach for the patients.

 ITM is a well-recognized but rare symptom of *Mycobacterium tuberculosis* infection. However, in developing countries, mycobacteria infection remains a significant and threatening problem. Most mycobacteria infections in humans result in asymptomatic, latent infections [4, 5]. So the index of suspicion for these infections must therefore be raised to a level in ITM. Open-label use of antituberculous treatment (ATT) was reported as beneficial in neuromyelitis optica (NMO) patients' refractory to immunotherapy [6]. Given the lack of proven efficacious treatments, this prospective, uncontrolled trial was designed to test ATT efficacy for treating ITM. We assessed patient's disability using a panel of clinical scales at baseline and after 1 or 2 years of ATT. Disease progression was followed by sequential MRI studies.

## 2. Patients and Methods

This prospective, open-label study was conducted at the First Affiliated Hospital of Sun Yat-sen University (Guangzhou, China), from Jan 2003 to Jun 2010. The Neurology Department in this hospital is a referral center focused on CNS diseases and accepts patients from other hospitals in southern china. Sixty-seven patients qualified for inclusion in the study, according to the inclusion and exclusion criteria (see below). There were 24 males and 43 females. The male to female ratio was 6 : 11. The median patient age was 39.07 ± 18.33 years. A detailed neurological examination was carried out, including evaluation of the motor, sensory, visual, and sphincter systems. A Mantoux test, chest X-ray, serum biochemistry, serum and cerebral spinal fluid (CSF) Borrelia, and human immunodeficiency virus (HIV) testing were performed to exclude other diseases. The CSF cell count, glucose, and protein levels were obtained from lumbar punctures performed prior to the onset of ATT. A direct smear for acid-fast bacillus and a *M. tuberculosis* antibody test (PPD-lgG) were also conducted. The presence of oligoclonal bands was recorded in 26 of the 67 patients. 

Upon admission, a spinal MRI was performed on all patients. The number of levels affected in the sagittal plane in the T2 sequence was measured. A brain MRI was also performed and if the MRI scan was not normal, the patient was excluded.

### 2.1. Inclusion and Exclusion Criteria

This study was conducted with patients in whom MTB was suspected clinically but not proven based on conventional measures. The inclusion criteria were (i) development of sensory, motor, or autonomic dysfunction attributable to the spinal cord, (ii) varying degrees of motor, sensory, and sphincter dysfunction (although not necessarily symmetric), but without complete paraplegia, (iii) exclusion of extra-axial compressive etiology by MRI, (iv) worsening symptoms despite at least one 5-day course of IV methylprednisolone (0.5–1 g/day), and (v) negative CSF MTB cultures with a cell count <50/mm^3^ and a total protein <1.5 g/L. The exclusion criteria were (i) sudden onset, (ii) history of previous radiation to the spine within the last 10 years, (iii) CNS manifestations of syphilis, Lyme disease, or HIV infection, (iv) clear arterial distribution and clinical deficit consistent with thrombosis of the anterior spinal artery, (v) history of clinically apparent optic neuritis, (vi) brain MRI abnormalities suggestive of MS or clinically definitive MS, and (vii) serologic or clinical evidence of connective tissue disease, such as, sarcoidosis, Behcet's disease, Sjögren's syndrome, systemic lupus erythematosus (SLE), or mixed connective tissue disorder.

The university ethics committee approved the study. All patients were informed of the potential short- and long-term drug complications of ATT. Written informed consent was obtained from patients who agreed to participate in the study. Patients were instructed to contact the study neurologist in the event of neurological symptoms. Before the start of ATT treatment, all patients received a baseline evaluation.

### 2.2. Study Drug Administration

Prior to ATT initiation, all treatments with corticosteroids and other systemic immunosuppression therapies were discontinued. Treatment protocols consisted of three antituberculous drugs (isoniazid, rifampicin, and pyrazinamide for 9 months), followed by a combination of isoniazid and rifampicin for 24 months. The doses were isoniazid at 10 mg/kg/day, rifampicin at 10 mg/kg/day, and pyrazinamide at 25 mg/kg/day. Treatment was under extensive observation. All patients had weekly liver function tests for the first one month of therapy and subsequently every 3 monthly (serum bilirubin, serum transaminases (SGOT/SGPT), and alkaline phosphatase).

### 2.3. Assessments

Eligible patients underwent complete physical and neurological examinations at entry, at 12 and 24 months, and as needed for assessment of acute relapses or safety. The American Spinal Injury Association (ASIA) standards [7] were adopted to assess subjects' neurological status. We used the ASIA Impairment Scale to evaluate sensory and motor function of the degree of spinal injury. Activities of daily living were assessed by the Barthel Index (BI) (0–100 scale, with lower scores denoting less independence in activities of daily living). Mobility was scored by the Hauser Ambulation Index (AI) [8] (0–9 scale designed to assess mobility). All patients were followed for at least 2 years after treatment. Quality of life changes were measured by the ASIA, BI and AI indices at baseline and at 24 months, along with evaluation by MRI. Each patient was followed up and assessed by the same physician during the study. 

Multiple sclerosis was diagnosed according to McDonald et al.'s criteria [9]. Patients that subsequently developed optic neuritis with or without recurrent ATM, but had no clinical manifestations or involvement of other parts of the CNS, were classified as NMO according to Wingerchuk et al.'s criteria [10].

### 2.4. Statistical Methods

Statistical analysis was performed using Student's *t*-test on paired samples. SPSS 13.0 statistical Analysis System software was used for statistical analysis.

## 3. Results

### 3.1. Demographic and Clinical Characteristics

The median interval from onset to treatment with ATT was 7.5 months (range, 1–57 months). The most common modality of presentation was in the form of sensory-motor involvement (59 patients, 88%). Motor impairment presented as asymmetric weakness, paraparesis or even quadriparesis. Sensory symptoms included numbness or paresthesias. Eight patients presented with just motor involvement (12%), whereas 26 patients (38.8%) had some form of urinary symptoms in the form of absent bladder sensations, incontinence, or increased urination frequency. Three patients had definitive TB evidence upon chest radiography (Mantoux test ≥ 10 mm in 48 hours). Baseline demographic and clinical information is reported in Table 1. 

The CSF findings were as follows. Elevated opening pressure (>200 mm H_2_O) was present in eight patients, and pleocytosis was found in 29 patients (43.3%) with lymphocyte predominance. Protein level was increased (>0.45 g/L) in 42 patients, 5 patients (7.5%) had decreased glucose concentration, and eight patients (11.9%) revealed a low CSF chloride level. Overall, CSF was abnormal in 55 of 67 patients, with 12 (17.9%) completely normal. 

Spinal MRI lesions were visible in 43 patients (64.2%). Lesions were usually hypointense or isointense on T1-weighted and hyperintense in T2-weighted sequences. In 14 cases, we observed obvious spinal cord swelling. DTPA-enhanced MRI scans were performed for 19 patients, of which 13 showed Gd-enhancement. Ring enhancement and/or heterogeneous enhancement were common findings. Two patients had associated syrinx. Brain MRI was performed for all patients but did not reveal any infectious or demyelinating lesions.

### 3.2. Followup

**Figure 1:**
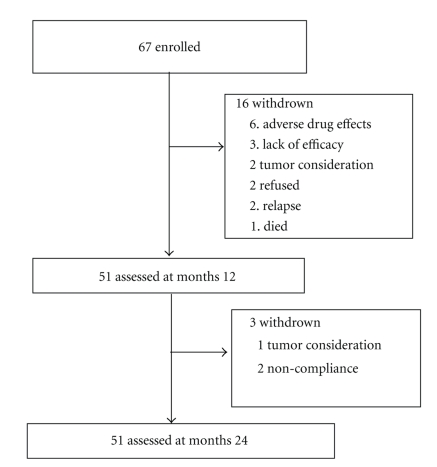
Trial profile.

Sixteen (23.9%) of 67 patients discontinued ATT within the first year. Another three patients dropped out during the second year medication but were still followed (Figure 1). Thus, complete 24-month data were available for 51 (76.1%) patients. The principle reasons for discontinuation were either side-effects or absence of perceived efficacy. Six patients withdrew early due to adverse drug effects. Three patients dropped out because of physician or participant's perception of therapy ineffectiveness. Two patients dropped out due to intramedullary spinal cord tumor considerations that required visiting another hospital. Two patients stopped treatment because of relapse, and two patients exhibited poor compliance. One patient died following an undiagnosed high fever and cerebral salt-wasting syndrome. The median follow-up interval after initial ATT was 32.8 months (range, 24–92 months).

### 3.3. Treatment Efficacy

Treatment was halted for patients experiencing deteriorating clinical symptoms since 43/51 (84%) patients experienced good response in the early course of ATT treatment. During the first week, 18/51 (35%) patients had considerable improvement in limb weakness. Sphincter symptoms gradually ameliorated in 21/26 (81%) patients. The clinical benefit was sustained and continued to improve in most cases. After one year, 49/51 (96%) of treated patients had achieved marked improvement of neurological signs, and five had experienced a full recovery without any motor or sensory sequelae. Spinal cord evaluation showed improved ASIA scores, significantly increased BI ratings, and an improved Hauser AI. The benefit was sustained and continued to improve over the second year (Table 2). However, the major improvement occurred during the first year of ATT treatment.

Neurological relapses were experienced by 9/51 (18%) patients, although they improved upon active treatment. During the first year followup, 13 relapses occurred, primarily in the initial three months (six relapses), with six more occurring in the second year of ATT treatment. Four patients who continued ATT treatment in relapses periods, and did not receive other immunosuppressive treatment, recovered, whereas five patients that received IVIG treatment responded well. Two patients experienced relapse of visual decline (at 6 months and 15 months, resp.) as limb weakness greatly improved, although both patients repeatedly showed a normal brain MRI. NMO diagnosis was established according to Wingerchuk's criteria. Similar to our previous study [6], visual acuity gradually recovered without change to our regimen. Eighteen relapses occurred in patients with longitudinal extensive lesions, and one relapse occurred in a patient involving less than two segments.

### 3.4. MRI Changes

**Figure 2:**
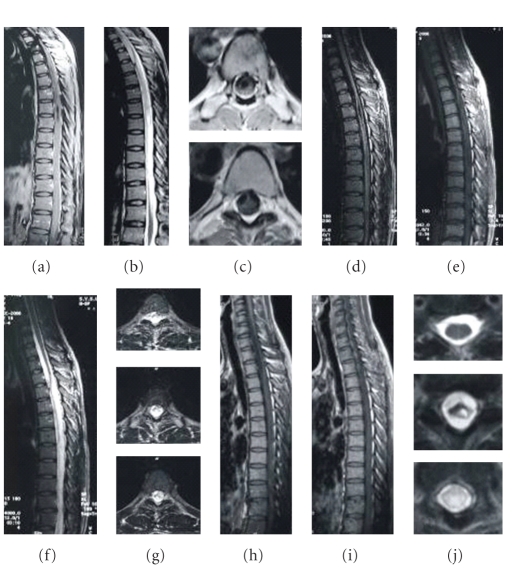
A 25-year-old woman was admitted due to the progressive weakness and parasthesia in both legs with voiding problem for 3 months. After 2-year ATT, her situation improved with good recovery of spinal function. The MRI changes are as follows: (a) first MRI performed in August 2005, T1 weighted image showing cord edema from T1 to bottom in the sagittal plane, with focal atrophy and syrinx cavity, (b) T2-weighted image showing a contiguous area of increased signal intensity, (c) cavity and band-like structure on axial T1-weighted images, (d, e) after one year of treatment, the swelling of cervical spinal cord greatly dissolved on sagittal T1-weighted image; the syrinx cavity is obviously diminished, (f) contiguous abnormal signal still left but diminished on the sagittal T2-weighted image, (g) the lesion is still obvious on axial T2-weighted images, (h, i) three years after treatment, the syrinx cavity is not obvious, and (j) some parts of thoracic cord restore normal signal on T2-weighted axial cord images. A small cavity is visible on T8, and increased signal intensity still exists below T9.

**Figure 3:**
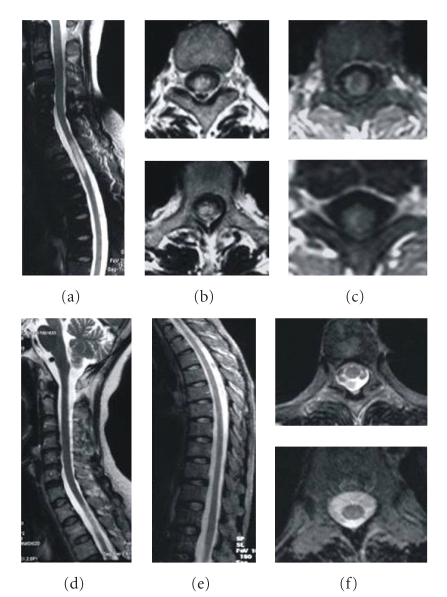
A 58-year-old female developed paraplegia and paresthesias in August 2007; weakness slowly worsened. Pulse methylprednisolone and IVIG were initiated with no resolution; she continued to deteriorate. After 2-year ATT, her situation fully recovered. The MRI changes are as follows: (a) sagittal T2-weighted image showing a contiguous area of increased signal intensity spanning T2 to T3 level, (b) axial T2-weighted images reveal the strong signals, (c) axial MRI images showing focal cord enhancement, (d) after 5 months of ATT, the lesion decreased, (e) following 1-year ATT, sagittal T2-weighted MRI of the thoracic cord reveals no abnormal signals, and (f) no lesion is visible on cross-sectional T2-weighted images.

**Figure 4:**
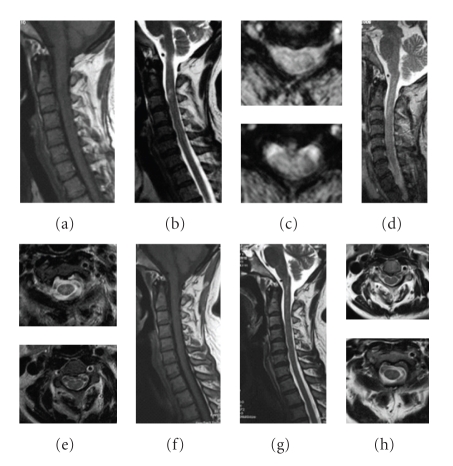
A 51-year-old female suffered from tetraparesis and frequently paroxysmal tics of extremities for 3 months. ATT treatment quickly improved her symptoms. After 2-year ATT, she fully recovered without visible symptoms. The MRI changes are as follows: (a) sagittal T1-weighted MRI showing focal thickening of the cord at C2-C3 level, (b) sagittal T2-weighted MRI showing a focal demyelinating lesion, (c) axial T2-weighted MRI showing the lesion to involve the whole cord, (d) sagittal T2-weighted MRI after 6 months of treatment, showing the swelling of the cervical spinal cord and patchy abnormal signal have resolved, (e) axial T2-weighted MRI showing an abnormal signal remains, (f) sagittal T1-weighted MRI after 2 years of treatment is normal, (g) sagittal T2-weighted MRI after 2 years of treatment revealing no abnormal signals, and (h) axial T2-weighted cord MRI after 2 years of treatment.

**Figure 5:**
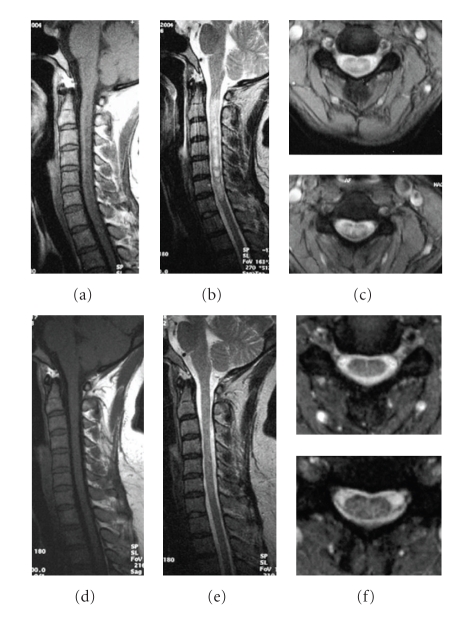
A 40-year-old woman had tetraparesis, C3 sensory level, and urinary incontinence for five months. Pulse methylprednisolone and IVIG were initiated with no resolution. However, ATT greatly improved her condition. The MRI changes are as follows: (a) sagittal T1-weighted MRI showing thickening of the cervical cord, (b) sagittal T2-weighted MRI showing a contiguous area of increased signal intensity spanning C2 to C7, (c) axial T2-weighted MRI showing the strong signal derived from the whole cord, (d) sagittal T1-weighted MRI after 2 years of treatment is normal, (e) sagittal T2-weighted MRI after 1 year of treatment, showing a normal spinal cord, and (f) axial T2-weighted MRI, showing no lesions.

**Figure 6:**
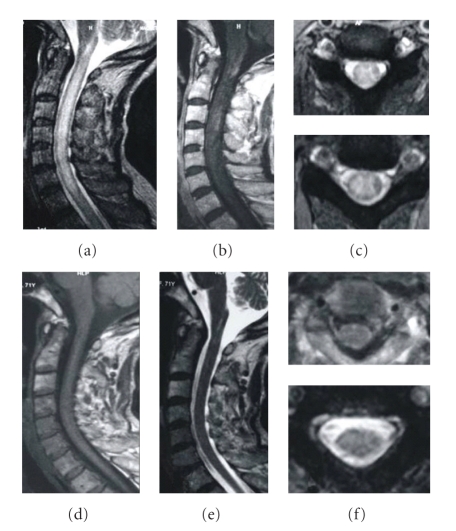
A 70-year-old female was admitted due to progressive paraparesis, paresthesias, and urinary retention for 2 months. After 2-year of ATT, her situation improved. The MRI changes are as follows: (a) sagittal T2-weighted image reveals a long, high intensity signal from C1 to T1 in the spinal cord before treatment regimen, (b) T1W contrast image showing patchy enhancement and thickening of the cord, (c) the diffuse lesions on axial T2-weighted images, (d) a year later the swelling of the cervical spinal cord disappeared on sagittal T1-weighted images, (e) the hyperintensity resolved following 1 year of ATT, and (f) no lesion is visible on axial T2-weighted images.

**Figure 7:**
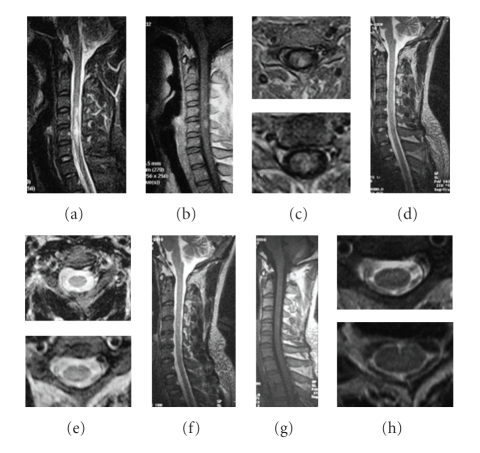
A 30-year-old male suffered from left paraparesis and paresthesias for 1 month. MRI of the cervical spinal cord revealed a demyelinating lesion in T2-weighted images extending from C4 to C7. Pulse methylprednisolone and IVIG were initiated with no resolution. The MRI changes are as follows: (a) sagittal T2-weighted image reveals a high intensity signal from C4 to C7 before ATT regimen, (b) sagittal T1-weighted MRI showing light thickening of the cord with subtle intraparenchymal hyperintensity, (c) the left local lesions on axial FLAIR sequences, (d) the hyperintensity resolved following 6 months of ATT, (e) no lesion is visible on axial T2-weighted images, and (f)–(h) no lesion is visible on follow-up MRI after 2 years of treatment.

**Figure 8:**
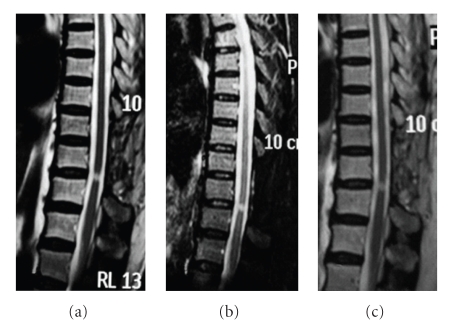
A 67-year-old female suffered from progressive paraparesis and paresthesias for 1 month. There is a small demyelinating lesion at the level between T9 and T10. Steroid pulsed treatment worsened her condition. After ATT, her weakness quickly improved. She tried to stop ATT two times during the early stage of treatment due to drug side-effects, but failed because her condition grew worse. Although she got better, the lesion seems to have not changed after 18 month treatment. (a) Sagittal T2-weighted image reveals a small high intensity signal between T9 and T10 before our regimen; (b) sagittal T2-weighted image after 6 months of ATT; (c) the lesion still exists on sagittal T2-weighted images after 18 months of treatment.

Two-year observations based on MRI imaging were completed for 33/51 (65%) patients who had cord lesions. Abnormal symptoms and swollen spinal cords progressively decreased in 32 patients after therapy initiation, and T2 signal abnormalities decreased markedly in size. This happened in poorly defined, extensively swollen cord lesions (Figure 2), with spinal cord demyelination involving less than two segments (Figures 3 and 4), and with longitudinal lesions involving more than three segments (Figures 5, 6, and 7). Additionally, regions where well-defined signals and poorly defined enhancements had been seen on pretreatment resolved with treatment. During the observational period, however, a decreased intramedullary lesion burden was not detected in two patients. One patient received an operation at 19 months due to lesion expansion, and pathological examination revealed an astrocytoma. The lesion remained in another patient with improved condition after 24-month followup (Figure 8).

### 3.5. Adverse Events

Adverse events occurred in 16/67 patients (23.9%). Two patients had somnolence and weakness due to hyponatremia during the first months of ATT treatment and quickly recovered on active therapy. No other serious adverse effects were seen but six patients withdrawn early because of lethargy, nausea, and vague ill health. Eight patients had mild gastrointestinal syndrome on active therapy but were able to continue the trial and resolved quickly. One death that occurred during the study was considered unrelated to the trial.

## 4. Discussion

We report our experience with a select group of myelopathy patients treated with ATT. This is the first clinical study to evaluate the therapeutic effects of ATT on steroid-refractory ITM. Sustained increases in ASIA scores and BI, with decreased AI scores, show that treatment is effective in most cases. This study used only pure antituberculosis chemotherapy, without adopting steroid and other immune suppressing drugs. The encouraging results are not only associated with significantly improving the neurological status of these patients, but also with beneficial changes to spinal lesions as determined by MRI. This study suggests ATT has beneficial effects in some ITM patients, and that *M. tuberculosis* infection may be an important cause of this disease.

Tuberculosis is still a major health problem in many parts of the world, especially Asia and Africa. However, tuberculous intramedullary involvement is considered to be very rare compared with tuberculous spondylitis or arachnoiditis [11]. Since its first description by Abercrombie in 1829 [12], there have only been some isolated cases reported. A true epidemiological and clinical profile has been elusive. This study shows mycobacterial infections might be more common than usually suspected in myelitis. However, due to the absence of a sensitive laboratory test, many cases may have been considered as “idiopathic” with a presumed immune mediated pathophysiological mechanism, despite extensive diagnostic workup. 

The etiology of the MRI signal abnormalities remains speculative. In the current series, the MRI signal changes in ITM vary from a small single lesion to extended longitudinal lesions over several spinal segments. In the literature, the majority of ITM cases with a single lesion is diagnosed as a clinically isolated syndrome (CIS), which later may be converted to multiple sclerosis (MS) [13, 14]. However, not all CIS patients develop MS. Another diagnostic problem is posed by patients with long hyperintense MRI lesions, involving three or more segments of the spinal cord, or the so-called longitudinal extensive transverse myelitis (LETM). Today, such patients are designated as part of the NMO spectrum [15]. In this study, CIS and LETM lesions were revealed gradually as the imaging resolution of the abnormalities improved after ATT therapy was initiated. The results of the present study suggest that lesion length may not correlate with different pathological findings. It is possible that aspects of LETM and CIS are based on the same mycobacteria etiology. However, this hypothesis may serve as a basis for further studies since this small series of patients and study design was not designed to address this important question. 

In this study, 24 patients (38.2%) develop ITM without signs of demyelination or other abnormalities of the spinal cord as determined by MRI. Previous reports [16] show patients in this group usually have worsening symmetrical clinical symptoms of transverse spinal cord syndrome, in which spinal MRI may show atrophic changes later. There is no evidence in the literature that any effective treatment exists. However, ATT shows an excellent treatment effect for 17 patients of this trial. It may be that these puzzling results are associated with mycobacteria infection. It is noteworthy that patients in this category may present with clinical symptoms similar to that of tropical spastic paraparesis (TSP), which has been associated with human T-cell lymphotropic virus type 1 (HTLV-1) [17]. TSP diagnosis may require exclusion by laboratory tests for HTLV-1 antibodies. Nevertheless, trial ATT treatments may be beneficial in tuberculosis endemic areas where diagnosis is not definite, but where no treatment is available to treat TSP patients.

We found that subsequent neurological relapses during ATT treatment usually occur in at least some patients, potentially through a mechanism similar to paradoxical deterioration in tuberculosis. Paradoxical deterioration during ATT is usually defined as the clinical or radiological deterioration of pre-existing tuberculous lesions or the development of new lesions in a patient who initially improved [18]. In three reported cases of acute myelopathy associated with pulmonary tuberculosis that have undergone postmortem examination [19], the myelopathy had been progressive or relapsing, and demyelination of the white and grey matter was found in the spinal cord of all three patients. Relapses in tuberculous myelopathy can recur several times during treatment [20]. Reid and Bone reported that clinical improvement can be achieved after steroid therapy [21]. However, disease progression despite adequate ATT treatment is well documented [22]. Although relapses occurred in some patients, there was a tendency for the relapse rate to decrease as treatment continued. In this trial, two LETM patients experienced optica neuritis, and hence satisfied criteria for a diagnosis of NMO. However, both patients successfully recovered without changing the regimen. 

There are still few reports describing the features of tuberculous myelopathy detected by MRI. The first documented description of using MRI to determine intramedullary spinal tuberculosis was published by Rhoton et al. in 1988 [23]. Lesions are usually hypointense on T1W images and iso-to-hyperintense on T2W images with cord expansion. Ramdurg et al. reported the largest series of 15 spinal intramedullary tuberculosis with varied MR changes [24]. This study shows excellent clinical outcomes can be obtained with a combination of medical treatment and surgical management, although patients presenting late had a poorer outcome. In our study, there was a significant effect of ATT treatment on T2-lesion volume over time. It is impressive that abnormal signals and expanded spinal cords progressively decreased with ATT and even completely disappeared in some cases. All these changes may just reflect that the infection subsided. Perhaps a more fundamental problem is related to the interpretation of results from ATT trials. Further studies should investigate whether this syndrome is due to an immune reaction to TB or a primary TB infection. 

There are methodological limitations to the current study. This is an uncontrolled study, the number of patients in the trial was small, and relapses were less well defined. Overall, the trial provides some inspiring data and indicates ATT may be an efficient, cost-effective approach to some patients. Based on this study, a double-blind controlled study of ATT efficacy will need to be performed, preferably over a longer duration and with a larger sample size.

## 5. Conclusion

We have presented a prospective pilot study on steroid-refractory ITM. This study has identified, for the first time, the long-term clinical efficacy of ATT in some steroid-refractory ITM patients. Our results suggest that ATT treatment can not only stop disease activity and progression but may also result in a significant recovery of fixed neurological deficits. On the other hand, the study also suggests that tuberculosis infection might be an important and still neglected cause of myelitis. Additional efforts must be made to conduct well-designed trials since no other effective treatments currently exist.

##  Financial Disclosure Statement

The authors did not have funding from any of the manufacturers.

## Figures and Tables

**Table 1:** Baseline demographic and clinical information at inclusion.

Characteristic	Treatment group
Number	67
Female/male	43/24
Age of study entry (years) mean ± SD	39.07 ± 18.33
Disease duration (months)	7.55 (range, 1–57)
Drop-out	16
Mantoux test *⩾* 10 mm.	48
Patients with CSF normal finding	12
Patients with abnormal MR change	43

**Table 2:** The changes of spinal function scores on baseline and after 1 year and 2 years of ATT (51 patients).

Years	ASIA sensory scores	ASIA motor scores	Barthel index	Ambulation index
Baseline	184.02 ± 23.57	78.86 ± 14.51	74.42 ± 22.40	3.92 ± 2.06
Year 1	209.33 ± 21.42*	93.75 ± 8.03*	93.43 ± 10.61*	1.63 ± 1.72*
Year 2	211.10 ± 19.96*	95.10 ± 8.34*	95.47 ± 6.25*	1.09 ± 1.36*

Each value represents mean ± SEM. *Improvement in values is statistically significant (*P* < .05) from baseline (paired sample *t*-test).
